# Can exercise truly alleviate mild-to-moderate and subthreshold depression? Evidence from randomized controlled trials

**DOI:** 10.3389/fpsyg.2026.1815160

**Published:** 2026-04-14

**Authors:** Duanying Li, Jun Chen, Tianyuan He, Ruixiang Yan, Wenfeng Zhang, Guoxing Li

**Affiliations:** 1 Key Laboratory of Human-Computer Intelligent Interaction for Athletic Performance and Health Promotion, Guangzhou Sport University, Guangzhou, Guangdong, China; 2 School of Athletic Training, Guangzhou Sport University, Guangzhou, Guangdong, China

**Keywords:** depression, exercise type, mild-to-moderate depression, physical activity, subthreshold depression

## Abstract

**Objective:**

This study centers on populations with mild-to-moderate and subthreshold depression, excluding pharmacological interference, to systematically evaluate the comparative efficacy of various exercise modalities.

**Methods:**

Adhering to PRISMA guidelines, a systematic search was conducted across PubMed, Embase, Web of Science, the Cochrane Library, and PsycINFO databases up to January 25, 2026. Inclusion criteria encompassed randomized controlled trials (RCTs) implementing structured exercise interventions for mild-to-moderate and subthreshold depression. A random-effects model was utilized to calculate the pooled effect size (Hedges’ *g*). Subgroup analyses were performed to explore the moderating effects of exercise type. Methodological quality was evaluated using the Cochrane RoB 2.0 tool.

**Results:**

A total of 37 RCTs (3,110 unique participants) were included, contributing 3,922 observations across multiple comparisons. The analysis revealed that exercise interventions significantly ameliorated symptoms of non-severe depression compared to control groups, yielding a large pooled effect size (Hedges’ *g* = −0.86, 95% CI [−1.06, −0.68], *p* < 0.001). High overall heterogeneity was observed (*I*^2^ = 82.0%). Subgroup analysis indicated differences in efficacy across exercise types: aerobic exercises (*g* = −0.91) and mind–body exercises (*g* = −0.88) demonstrated the strongest improvement effects; resistance training (*g* = −0.84) followed; and mixed/other exercises showed a moderate effect size (*g* = −0.64). Notably, mind–body exercises exhibited the lowest relative heterogeneity (*I*^2^ = 54%), suggesting superior therapeutic stability. Egger’s test indicated potential publication bias (*p* < 0.05).

**Conclusion:**

Physical exercise serves as an effective intervention for ameliorating non-severe depression. Among exercise modalities, aerobic exercise demonstrated the most substantial symptom alleviation effects, while mind–body exercises exhibited optimal therapeutic stability. However, the overall certainty of evidence is rated as ‘Low’ (GRADE), and the presence of potential publication bias necessitates a cautious interpretation of the exact effect size magnitude. Clinicians are encouraged to integrate structured exercise as a valuable adjunctive strategy for non-severe depression management.

## 1 Introduction

A substantial body of evidence from systematic reviews indicates that regular physical exercise, as a cost-effective non-pharmacological intervention, exerts a definitive ameliorative effect on depressive symptoms. Particularly within populations suffering from mild-to-moderate and subthreshold depression, exercise interventions have demonstrated immense potential for application. Depression represents a leading cause of global disability, affecting an estimated 280 million people worldwide, with mild-to-moderate and subthreshold forms contributing to a massive public health burden. Crucially, depression is strongly associated with suicidal behavior, and epidemiological studies have demonstrated that suicide attempts frequently occur during specific high-risk periods of the day and week, emphasizing the need for timely preventive strategies and effective non-pharmacological interventions such as structured exercise programs (Kanani and Sheikh, 2024). Previous high-quality randomized controlled trials (RCTs) suggest that for such populations, the short-term efficacy of exercise interventions is comparable to that of first-line antidepressants (e.g., Sertraline) (Brenes et al., 2007) or Cognitive Behavioral Therapy (CBT) (Hallgren et al., 2015). Given the vast size of the non-severe depression demographic and the fact that they are situated in a critical “window of opportunity” to prevent progression to Major Depressive Disorder (MDD), this highly accessible, low-side-effect intervention holds significant clinical value and public health implications. Despite the widespread confirmation of exercise’s overall effectiveness, the core challenge in clinical practice remains the formulation of “precision” exercise prescriptions. While existing research validates the efficacy of various exercise modes, a consensus regarding “which exercise type is superior” and the “optimal dose-response relationship” is lacking. Furthermore, the mechanisms underlying different exercise modalities may differ significantly. For instance, aerobic exercise is widely proven to improve cognitive control and emotional regulation by enhancing cardiorespiratory fitness, promoting neuroplasticity, and up-regulating Brain-Derived Neurotrophic Factor (BDNF) (Olson et al., 2017; Philippot et al., 2022). Conversely, resistance training may specifically target anhedonia and feelings of helplessness in depressed patients by increasing muscle strength, thereby elevating self-efficacy and a sense of mastery (Kratzer et al., 2021; Singh et al., 1997; Singh et al., 2005). Additionally, mind–body exercises represented by Yoga and Tai Chi have accumulated robust evidence for regulating autonomic nervous system balance, reducing cortisol levels, and alleviating comorbid anxiety (de Manincor et al., 2016; Liu et al., 2015; Zhang et al., 2022). Regarding intensity, literature supports the efficacy of a spectrum of interventions ranging from low-intensity mind–body regulation to High-Intensity Interval Training (HIIT) (Duan et al., 2025; Singh et al., 2005); however, whether high-intensity stimulation is superior or low-to-moderate intensity offers better adherence for non-severe depression populations remains inconclusive. The persistence of this “undefined prescription” status is largely constrained by the methodological limitations of previous evidence syntheses. Many meta-analyses tend to include data from a large number of MDD patients who are often simultaneously receiving standard antidepressant medication (Krogh et al., 2009; O'Sullivan et al., 2023). The “floor effect” created by pharmacological treatment not only masks the specific efficacy of exercise as an independent intervention but also makes it difficult for researchers to precisely isolate subtle efficacy differences between various exercise elements (type, intensity, frequency). Consequently, there is a scarcity of high-quality evidence specifically evaluating exercise as a monotherapy or core intervention for the specific population of non-severe depression.

## 2 Methods

### 2.1 Study protocol and literature search

**Figure 1:**
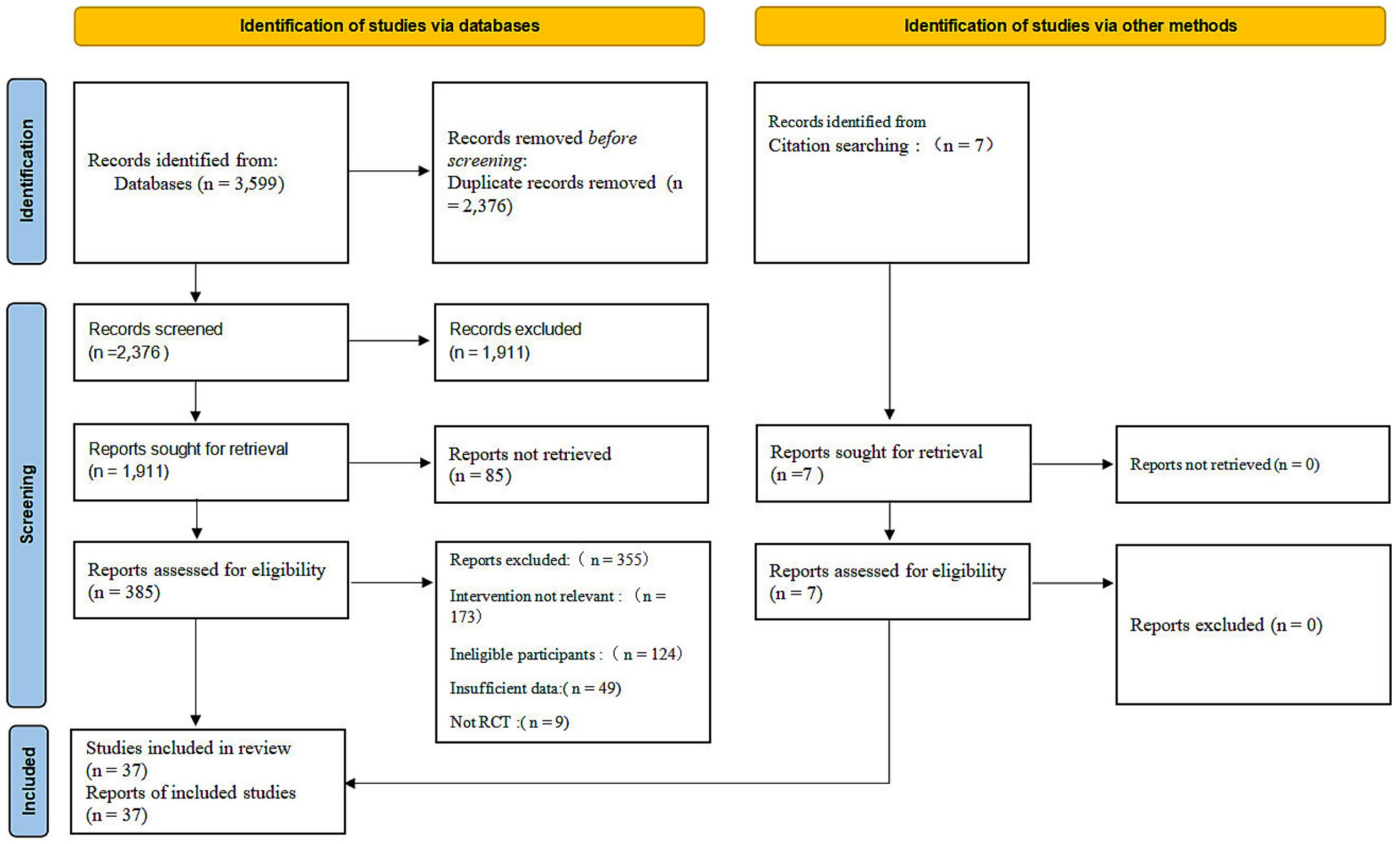
Literature screening flow diagram.

This study was conducted and reported in strict adherence to the *Preferred Reporting Items for Systematic Reviews and Meta-Analyses* (PRISMA) guidelines and has been registered with the PROSPERO International Prospective Register of Systematic Reviews. To comprehensively identify relevant literature, we systematically searched the PubMed, Embase, The Cochrane Library, Web of Science, and PsycINFO databases from their inception to February 2026. The search strategy employed a combination of Medical Subject Headings (MeSH) and free-text terms, encompassing English keywords such as “depression” (depressive disorder), “exercise” (physical activity), “aerobic,” “resistance training,” “mind–body exercise” (yoga, tai chi), and “randomized controlled trial.” The detailed process of literature screening is illustrated in Figure 1; the PRISMA 2020 Flow Diagram depicts the selection process from initial database retrieval to final inclusion in the analysis. The full search syntax for all databases is provided in Supplementary File S2 to ensure reproducibility.

### 2.2 Inclusion and exclusion criteria

Literature selection was based on pre-defined PICOS principles. The inclusion criteria were as follows:

(1) *Study design*: Published Randomized Controlled Trials (RCTs). (2) *Participants*: Adults and adolescents diagnosed with non-severe depression (including mild-to-moderate and subthreshold depression), excluding those with psychotic symptoms. (3) *Interventions*: The experimental group received structured physical exercise interventions. “Structured” refers to physical activity plans adhering to the FITT principles (Frequency, Intensity, Time, Type), characterized by explicit planning, repetition, and specific objectives. This includes aerobic exercise, resistance training, mind–body exercises (e.g., Yoga, Tai Chi), or mixed exercise modalities. General physical activity advice without a specific execution plan was not included. Where applicable, exercise intensity was classified into light, moderate, or vigorous categories based on standard objective markers such as the Metabolic Equivalent of Task (METs), the percentage of maximum heart rate (HRmax), or maximal oxygen uptake (VO2max) reported in the original trials. (4) *Comparators*: Control groups received usual care, waitlist, no intervention, or attention control. (5) *Outcomes*: Provided data necessary to calculate effect sizes for depressive symptoms (e.g., mean, standard deviation, and sample size).

Exclusion criteria included studies focusing on the acute phase of Major Depressive Disorder (MDD) or treatment-resistant depression, non-randomized or quasi-randomized trials, studies from which data could not be extracted, and duplicate publications derived from the same dataset.

### 2.3 Data extraction

Literature screening and data extraction were performed independently by two researchers (Chen Jun and He Tianyuan). Extracted data included study characteristics (author, year, country), participant demographics (age, gender, depression severity), specific intervention parameters (exercise type, intensity, frequency, session duration, total duration), and outcome measurement tools.

In the data pre-processing stage, given that the included studies utilized different continuous variable measurement tools, this study standardized the effect size metric using the Standardized Mean Difference (SMD) and calculated Hedges’ g along with its 95% Confidence Interval (CI) to correct for small sample bias. To ensure analytical consistency, the directionality of all outcome data was unified; specifically, score reductions were set to represent improvement in depressive symptoms, and signs were flipped for scales where high scores indicate better health (e.g., quality of life scores). Furthermore, for data where Standard Deviation (SD) was not directly reported, Standard Error (SE) or Confidence Intervals were converted to SD according to the Cochrane Handbook formulas. For missing SDs of change scores, calculations were based on the conservative estimation formula for correlation coefficients:


$${\mathrm{SD}}_{\text{change}}=\sqrt{{\mathrm{SD}}_{\mathrm{pre}}^{2}+{\mathrm{SD}}_{\text{post}}^{2}-(2\times r\times {\mathrm{SD}}_{\mathrm{pre}}\times {\mathrm{SD}}_{\text{post}})}$$

For multi-arm trials comprising multiple intervention groups, the control group sample size was divided equally among the respective intervention arms. This approach was adopted to prevent double-counting in the pooled analysis, thereby ensuring the statistical rigor and robustness of the meta-analytic results.

### 2.4 Risk of Bias and quality assessment

Methodological quality was assessed using the Cochrane Collaboration’s recommended Risk of Bias tool version 2.0 (RoB 2.0). Evaluation focused on five domains: randomization process, deviations from intended interventions, missing outcome data, measurement of the outcome, and selection of the reported result. The certainty of evidence was graded using the GRADE (Grading of Recommendations Assessment, Development and Evaluation) framework. Any discrepancies arising during screening, extraction, or assessment were resolved through consultation with a third senior researcher (Li Duanying).

### 2.5 Statistical analysis

All statistical analyses were conducted within the R language environment (Version 4.5.2), primarily utilizing the meta and metafor packages. Given the potential clinical heterogeneity in population characteristics (e.g., age, disease duration) and intervention protocols (e.g., type, duration) among the included studies, a Random-effects model based on the DerSimonian-Laird (DL) method for estimating Tau-squared (*T*^2^) was adopted for data pooling. The effect size metric selected for continuous variables was the Standardized Mean Difference (SMD), and Hedges’ g along with its 95% Confidence Interval (CI) was calculated to correct for potential bias arising from small sample sizes.

Heterogeneity between studies was assessed using Cochran’s *Q* test (significance level *α* = 0.10) and the *I*^2^ statistic, where an *I*^2^ value > 50% was considered indicative of significant heterogeneity. To explore sources of heterogeneity and the impact of different intervention characteristics, we pre-specified subgroup analyses based on exercise type (aerobic, resistance, mind–body, other) and exercise intensity (light, moderate, vigorous), calculating the test for subgroup differences (Qb). Additionally, a Leave-one-out sensitivity analysis was employed to verify the robustness of the pooled results, and potential publication bias was quantitatively assessed using Egger’s linear regression test and the Trim-and-fill method. Except for heterogeneity tests, all statistical inferences were two-sided, with *p* < 0.05 considered statistically significant.

## 3 Results

### 3.1 Literature selection process and characteristics of included studies

The literature search and screening process were conducted in strict accordance with the PRISMA guidelines (refer to Figure 1). The initial database search yielded a total of 3,599 relevant records. Following the manual removal of duplicates and preliminary screening of titles and abstracts, the full texts of the remaining 385 potentially eligible articles were reviewed. For articles where full texts were unavailable via institutional repositories or key data were missing, attempts were made to contact the corresponding authors via email (with a minimum of two data requests sent). After strict adherence to the inclusion and exclusion criteria, and the exclusion of studies with no response or unavailable data, 37 randomized controlled trials (RCTs) were ultimately included for quantitative synthesis.

**Figure 2:**
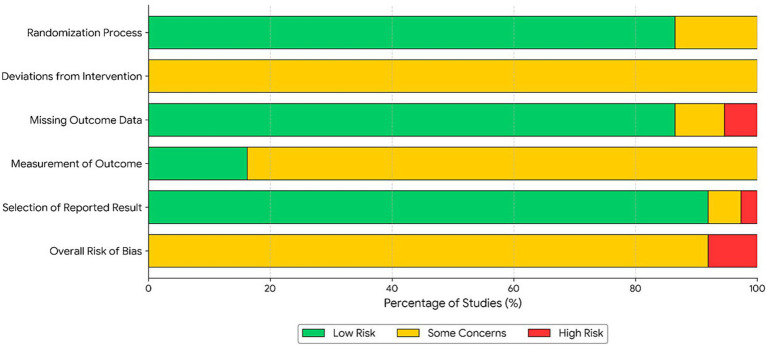
Summary of risk of bias assessment for included studies.

The included studies comprised a total of 3,110 unique participants. Notably, due to the splitting of control groups in multi-arm trials, these contributed to 3,922 observations across multiple comparisons. Sample sizes exhibited significant variation, ranging from single-arm sizes of 8 (Duan et al., 2025; Legrand and Heuze, 2007) to 300 (Hallgren et al., 2015). The study population spanned the entire life course, demonstrating robust external validity; it included multiple studies targeting adolescents (e.g., Gu et al., 2024; Nabkasorn et al., 2006), adults (e.g., Hallgren et al., 2015), and the elderly (e.g., Lu et al., 2020; Singh et al., 1997). Baseline depression levels were confirmed via standardized scales (e.g., HAM-D, BDI, CES-D) as mild-to-moderate or subthreshold, while patients with Major Depressive Disorder (MDD) currently undergoing antidepressant pharmacotherapy were explicitly excluded. The baseline characteristics of the included studies are summarized in Table 1. Intervention protocols exhibited significant diversity, reflecting the broad adaptability of exercise therapy. Regarding the distribution of exercise types, aerobic exercise was the most prevalent (18 studies), including jogging (Nabkasorn et al., 2006), swimming (Massey et al., 2025), and cycling. This was followed by mixed/other exercises (8 studies), encompassing High-Intensity Interval Training (HIIT) (Duan et al., 2025; Philippot et al., 2022), ball games (Li et al., 2025), and dance therapy (Hyvönen et al., 2020; Jeong et al., 2005). Mind–body exercises (7 studies) such as Yoga (de Manincor et al., 2016; Javnbakht et al., 2009; Woolery et al., 2004), Tai Chi (Liu et al., 2015; Zhang et al., 2022), and Qigong (Lu et al., 2020; Phansuea et al., 2020), as well as resistance training (4 studies) (Chen et al., 2023; Krogh et al., 2009; Singh et al., 1997; Singh et al., 2005), also constituted a significant proportion. Intervention durations were predominantly clustered between 8 and 12 weeks, with frequencies mostly ranging from 2 to 3 sessions per week. Notably, certain recent studies (e.g., Zhang et al., 2025) incorporated digital health technologies such as VR cycling (Zhang et al., 2025), reflecting emerging trends within the field. Figure 2 illustrates the network of evidence for the interventions in the included studies. Node sizes correspond to the sample size weight of the specific intervention type, while the thickness of the connecting lines represents the number of studies containing direct comparisons. The analysis indicates that Aerobic Exercise occupies the central position within the network, exhibiting the strongest direct evidence linkage with Usual Care (Control/TAU), thereby providing a robust comparative foundation for the network meta-analysis.

**Table 1:** Characteristics of included studies.

Study	Country	Population	Mean age (y)	Sample (I/C)	Intervention protocol	Control group	Outcome	Baseline
Adolescents								
Carter et al. (2015)	UK	Adolescents	15.4	44/43	Aerobic, 1x/wk, 12 s	TAU	CDI-2	Moderate
Chang et al. (2025)	China	Adolescents	13	80/80	Aerobic, 5x/wk, 45 m, 12w	Low Int.	CES-D	Moderate
Gu et al. (2024)	China	Adolescents	16.7	31/32	Aerobic (Open), 3x/wk, 40 m, 8w	Control	CES-D	Subthres
Jeong et al. (2005)	Korea	Adolescents	16	20/20	Dance (DMT), 3x/wk, 45 m, 12w	Control	SCL-90	Mild
Li et al. (2025)	China	Adolescents	13.4	29/21	Basketball, 3x/wk., 40 m, 8w	Control	CES-D	Moderate
Nabkasorn et al. (2006)	Thailand	Adolescents	18.8	25/24	Aerobic (Jogging), 5x/wk, 50 m, 8w	Usual Care	CES-D	Mild-Mod
Zhang et al. (2021)	China	Adolescents	15	66/69	Aerobic (Run/Walk), 4x/wk, 45 m, 16w	Medication	HAM-D	Moderate
Adults								
Haller et al. (2018)	Germany	Adults	38	10/10	Web-Exercise, Individual, 8w	TAU	QIDS	Moderate
Hallgren et al. (2015)	Sweden	Adults	43	300/300	Aerobic, 3x/wk, 60 m, 12w	TAU	MADRS	Mild-Mod
Helgadóttir et al. (2016)	Sweden	Adults	42.6	106/310	Mixed/HIIT, 3x/wk, 60 m, 12w	TAU	MADRS	Mild-Mod
Hyvönen et al. (2020)	Finland	Adults	39	52/57	Dance (DMT), 2x/wk, 60 m, 12w	TAU	BDI	Moderate
Kratzer et al. (2021)	Germany	Adults	41.6	70/70	Bouldering, 1x/wk, 3 h, 10w	CBT	MADRS	Moderate
Krogh et al. (2009)	Denmark	Adults	38.9	55/55	Resistance, 3x/wk, 60 m, 16w	Relaxation	HAM-D	Moderate
Legrand and Heuze (2007)	France	Adults	39.2	8/7	Aerobic, 3-5x/wk, 8w	Low Freq	BDI-II	Moderate
Liu et al. (2015)	Australia	Adults (Obese)	54	100/100	Tai Chi, 3x/wk, 60 m, 24w	Waitlist	DASS	Moderate
Luttenberger et al. (2015)	Germany	Adults	43.9	22/25	Bouldering, 1x/wk, 3 h, 8w	Waitlist	BDI-II	Moderate
Massey et al. (2025)	UK	Adults	42.6	43/44	Swimming, 1x/wk, 8 s	Usual Care	PHQ-9	Mild-Mod
Olson et al. (2017)	USA	Adults	21.1	15/15	Aerobic, 3x/wk, 30 m, 8w	Stretching	BDI-II	Moderate
Stelzer et al. (2018)	Germany	Adults	43.5	23/24	Bouldering, 1x/wk, 3 h, 8w	Waitlist	SCL-90	Moderate
Woolery et al. (2004)	USA	Young Adults	21	14/14	Yoga (Iyengar), 2x/wk, 60 m, 5w	Waitlist	BDI	Mild
Zhang et al. (2025)	China	Adults	39	33/34	VR Cycling, 3x/wk, 30 m, 12w	Cycling	HAM-D	Mild-Mod
de Manincor et al. (2016)	Australia	Adults	48.5	47/54	Yoga, 1x/wk, 6w	Waitlist	DASS	Mild-Mod
Elders								
Brenes et al. (2007)	USA	Elders	74	12/12	Aerobic, 3x/wk, 60 m, 16w	Usual Care	HAM-D	Minor
Chen et al. (2023)	USA	Elders	77.7	14/14	Resistance, 2x/wk, 45 m, 12w	Waitlist	GDS	Mild
Duan et al. (2025)	China	Elders	67	8/8	Mixed/HIIT, 3x/wk, 40 m, 16w	Workshop	GDS	Mild-Mod
Lu et al. (2020)	China	Elders	69.5	15/15	Qigong, 3x/wk, 60 m, 12w	Control	GDS	Mild
McNeil et al. (1991)	Canada	Elders	72.5	10/10	Walking, 3x/wk, 6w	Social	BDI	Moderate
Moraes et al. (2020)	Brazil	Elders	65	14/13	Aerobic, 2x/wk, 60 m, 12w	Control	HAM-D	Mod-Severe
Phansuea et al. (2020)	Thailand	Elders	75	33/33	Qigong, 3x/wk, 50 m, 12w	Control	TGDS	Mild-Mod
Singh et al. (1997)	USA	Elders	71.3	16/16	Resistance, 3x/wk, 45 m, 10w	Attention	BDI	Moderate
Singh et al. (2005)	Australia	Elders	68	30/30	Resistance, 3x/wk, 60 m, 8w	GP Care	HAM-D	Major/Minor
Special populations								
Javnbakht et al. (2009)	Iran	Women	32.5	34/31	Yoga, 2x/wk, 90 m, 8w	Waitlist	BDI	Mild
Philippot et al. (2022)	Belgium	Students	20.8	13/15	HIIT, Online, 8w	Control	DASS-21	Mild-Mod
Yun et al. (2025)	China	Univ. Students	20.5	20/21	Taekwondo, 2x/wk, 90 m, 8w	Control	BDI-II	Mild
Zeng et al. (2025)	China	Students	18.5	24/24	Aerobic (MICT), 3x/wk, 45 m, 12w	Sedentary	SDS	Mild
Zhang et al. (2022)	China	Middle-aged	50.6	20/19	Tai Chi, 2x/wk, 90 m, 12w	Waitlist	HAM-D	Moderate
Zhao et al. (2022)	China	Students	21.2	29/28	Aerobic, 3x/wk, 45 m, 12w	Waitlist	SDS	Mild-Mod

Baseline depression severity (e.g., “Mild-Mod,” “Subthreshold”) was strictly classified according to the World Health Organization (WHO) diagnostic criteria for depressive episodes. The respective scale score thresholds reported in the included original trials were cross-referenced with WHO guidelines to ensure accurate and standardized categorization. Studies including patients with severe Major Depressive Disorder (MDD) or those currently undergoing concurrent pharmacotherapy were systematically excluded.

### 3.2 Risk of bias assessment

The assessment results, utilizing the Cochrane Risk of Bias tool version 2.0 (RoB 2.0), are illustrated in Figure 2. The included studies demonstrated favorable performance in the “Randomization Process” domain, with all trials employing appropriate methods for random sequence generation. However, due to the inherent nature of non-pharmacological behavioral interventions, all studies were categorized as raising “Some Concerns” in the domain of “Deviations from Intended Interventions,” primarily due to the infeasibility of blinding participants to the intervention.

**Figure 3:**
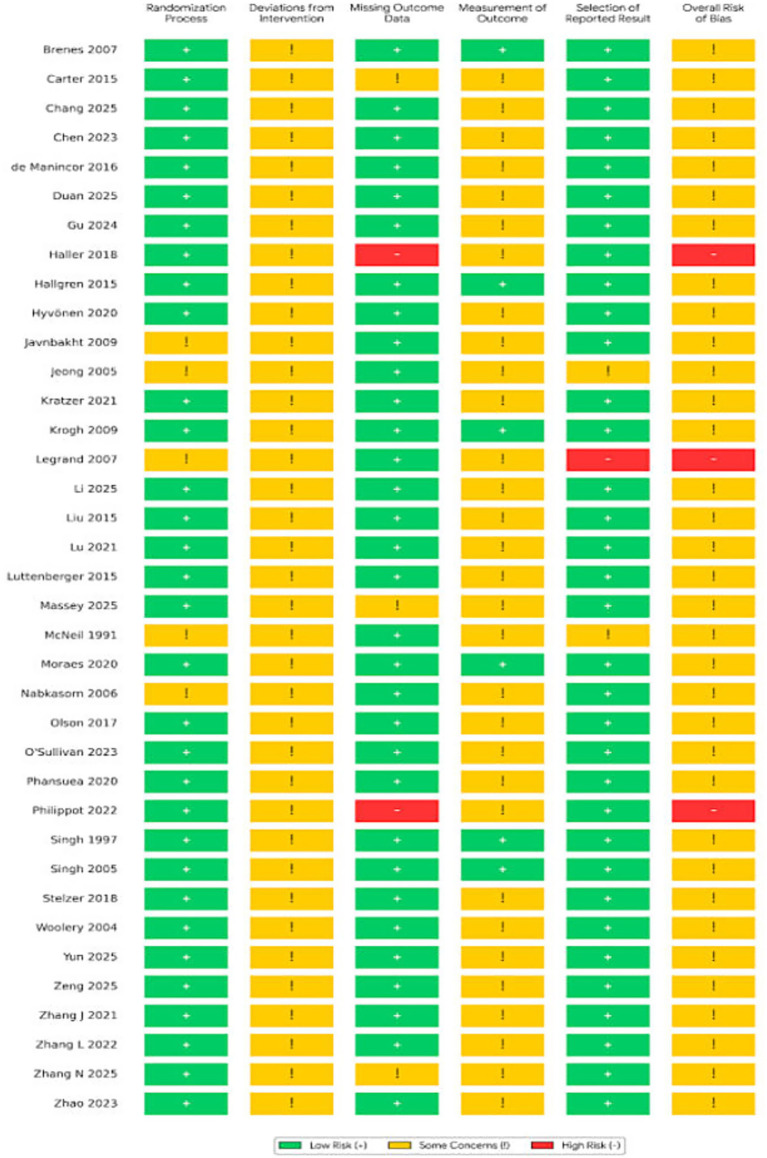
Summary of risk of bias assessment for included studies (traffic light plot).

Regarding outcome measurement, approximately 20% of the studies employed blinded assessors (e.g., clinician-rated HAM-D), presenting a lower risk of bias; conversely, the remaining studies, which relied on self-report scales (e.g., BDI, GDS), were subject to potential measurement bias. Notwithstanding these limitations, given the high degree of data integrity and standardized reporting, the overall quality of evidence was deemed suitable for meta-analysis. The detailed risk of bias assessment results are presented in Figures 2, 3.

### 3.3 Overall efficacy of exercise intervention on non-severe depression

Results from the meta-analysis, employing a random-effects model, demonstrate that exercise interventions significantly ameliorated depressive symptoms in patients with non-severe depression when compared to control groups (comprising usual care, waitlist, or no intervention). Synthesizing effect size data from 37 independent studies, the pooled effect size (Hedges’ g) was calculated to be −0.86 (95% CI [−1.049, −0.670], Z = −8.89, *p* < 0.001).

**Figure 4:**
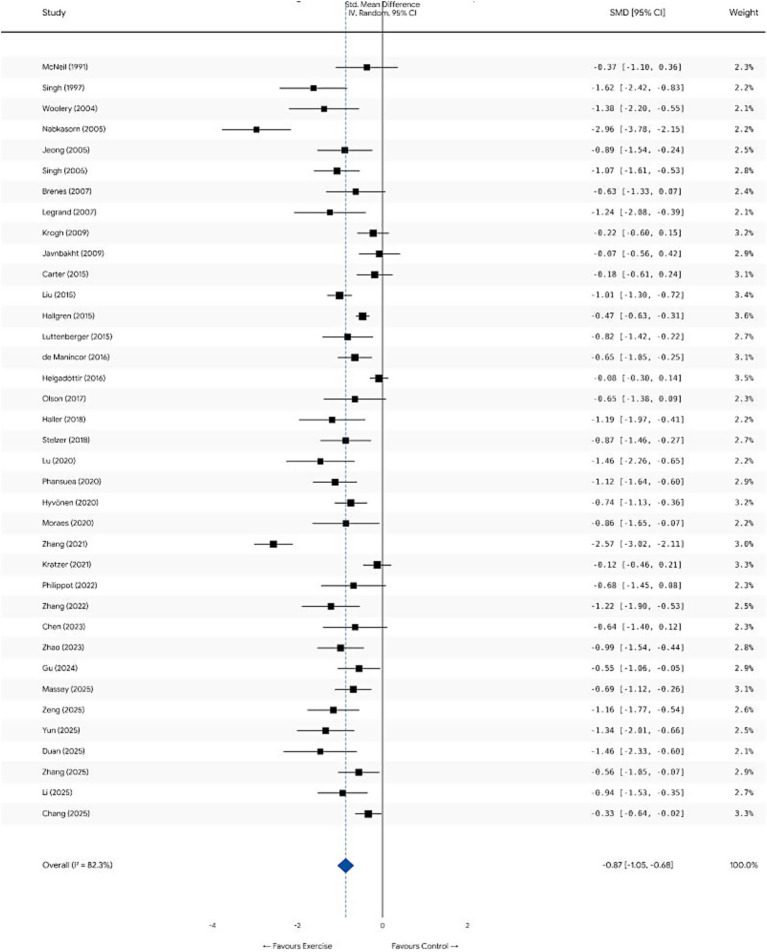
Forest plot (overall efficacy).

According to Cohen’s benchmarks for effect sizes (where 0.2 represents a small effect, 0.5 a medium effect, and 0.8 a large effect), this result indicates that exercise intervention exerts a clinically significant improvement on non-severe depression, characterized by a large effect size. Significant high heterogeneity was observed across studies (*Q* = 200.15, df = 36, *p* < 0.001; *I*^2^ = 82.0%). This suggests the presence of potential moderating variables influencing efficacy—such as participant demographics, exercise modalities, and intensity levels—warranting further exploration through subsequent subgroup analyses. The forest plot depicting overall efficacy is presented in Figure 4.

### 3.4 Heterogeneity analysis and subgroup analysis

**Figure 5:**
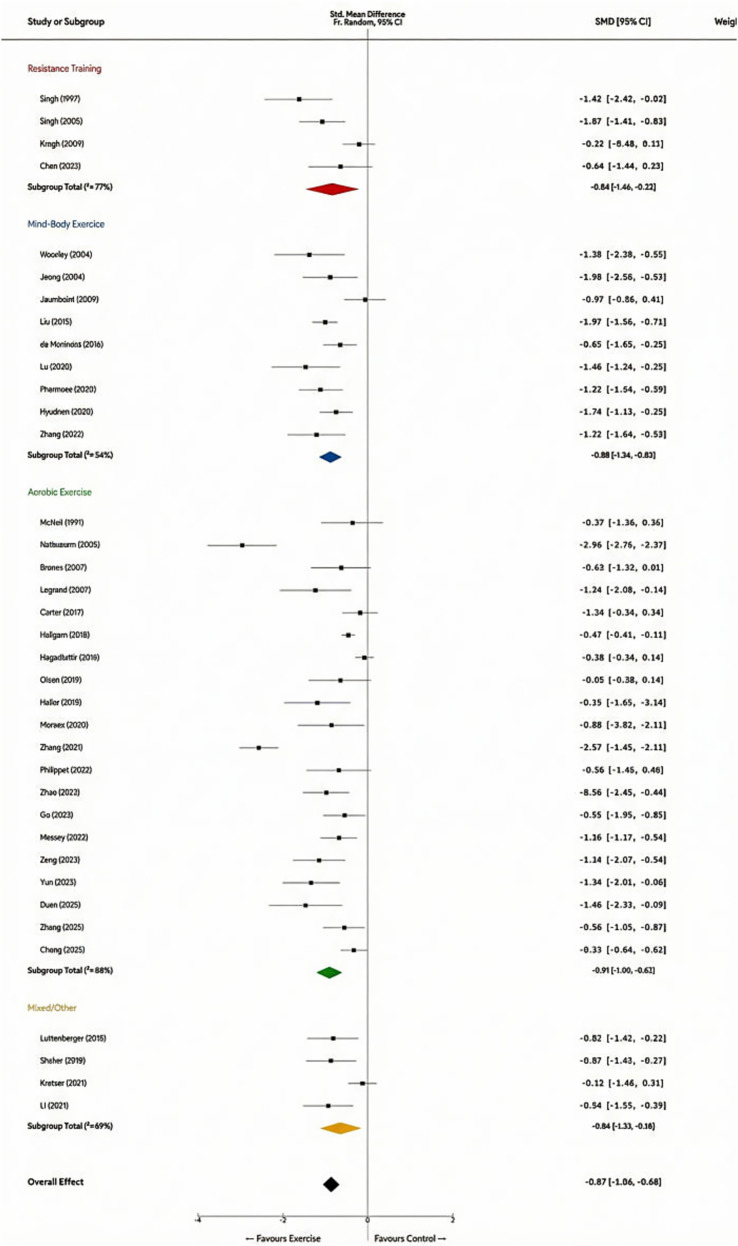
Subgroup analysis forest plot.

The overall analysis revealed significant high heterogeneity across studies (*I*^2^ = 82.0%, *Q* = 200.15, *p* < 0.001). To investigate the sources of heterogeneity and the efficacy characteristics of different intervention modalities, we conducted a prespecified subgroup analysis based on exercise type. The test for subgroup differences indicated that the variation in efficacy between different exercise types was statistically significant (*p* < 0.05). The specific results of the subgroup analysis are as follows (see Figure 5): Aerobic Exercise: As the most extensively studied intervention (*k* = 18), it showed the strongest intervention effect (*g* = −0.91, 95% CI [−1.20, −0.61]) (Hallgren et al., 2015; Olson et al., 2017; Zeng et al., 2025). However, the internal heterogeneity within this group was high (*I*^2^ = 88%), which may be attributed to differences in specific exercise forms (e.g., running vs. swimming) and intensity settings.

Mind–Body Exercise: Interventions such as Yoga, Tai Chi, and Qigong demonstrated a remarkably similar large effect size (*g* = −0.88, 95% CI [−1.34, −0.63]) (de Manincor et al., 2016; Liu et al., 2015; Zhang et al., 2022). Notably, the heterogeneity within this group (*I*^2^ = 54%) was distinctly lower than that of the aerobic exercise group, indicating that as a gentle intervention, its therapeutic efficacy is relatively stable. Resistance Training: This modality also exhibited a robust large effect size (*g* = −0.84, 95% CI [−1.46, −0.23]). Although the effect size was slightly lower than the former two, it remains highly effective and suggests that the results possess high consistency and replicability within specific populations, such as the elderly (Chen et al., 2023; Singh et al., 1997; Singh et al., 2005). Its internal heterogeneity remained relatively high (*I*^2^ = 77%). Mixed/Other Exercise: Comprising High-Intensity Interval Training (HIIT) and ball games, this group showed a moderate-to-large effect size (*g* = −0.64, 95% CI [−1.10, −0.18]) (Duan et al., 2025; Li et al., 2025; Philippot et al., 2022). Although slightly lower than the top three groups, it remains of distinct clinical significance.

Furthermore, to address the potential inflation of effect sizes by waitlist designs, a sensitivity analysis stratifying by control group type was performed. The active control group maintained a significant and robust effect size (SMD = −0.96, *p* < 0.0001), which approximates the effect size observed in the waitlist group (SMD = −1.07). Additionally, a meta-regression was conducted to explore the substantial heterogeneity, revealing that neither participant mean age (Coefficient = 0.004, *p* = 0.47) nor intervention duration (Coefficient = −0.048, *p* = 0.13) significantly moderated the treatment effects (*R*^2^ = 0.00%), suggesting consistent benefits across different ages and typical program lengths.

### 3.5 Sensitivity analysis

**Figure 6:**
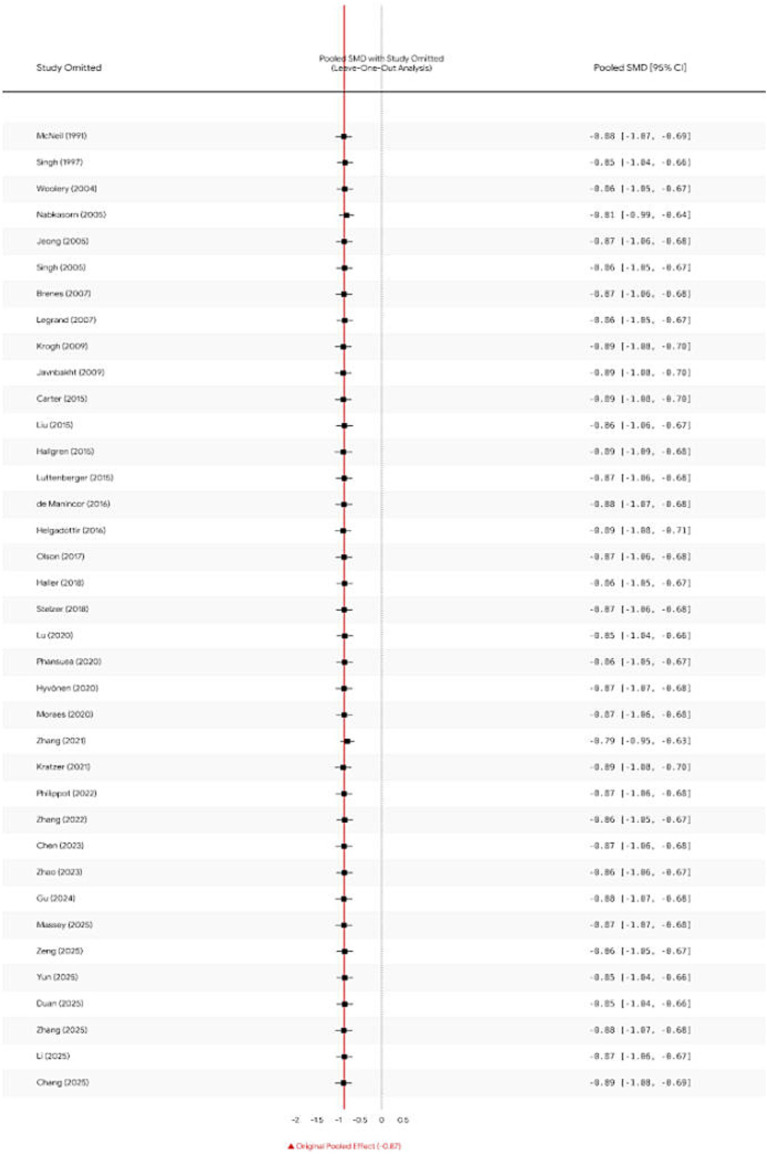
Sensitivity analysis (leave-one-out forest plot).

To verify the robustness of the primary results, a Leave-One-Out sensitivity analysis was conducted. As illustrated in Figure 6, following the sequential exclusion of individual studies, the recalculated pooled effect sizes (Hedges’ g) exhibited marginal fluctuations ranging from −0.842 to −0.875. Crucially, none of the recalculated 95% confidence intervals spanned the null value (zero). This finding indicates that the overall effect size (−0.859) possesses exceptional stability and was not unduly influenced by any single outlier or large-scale study (e.g., Hallgren et al., 2015). Furthermore, a comparison of computational results derived from different statistical models (DerSimonian-Laird vs. REML) revealed negligible differences in effect size (Δ*g* < 0.01), further corroborating the reliability of the findings.

**Figure 7:**
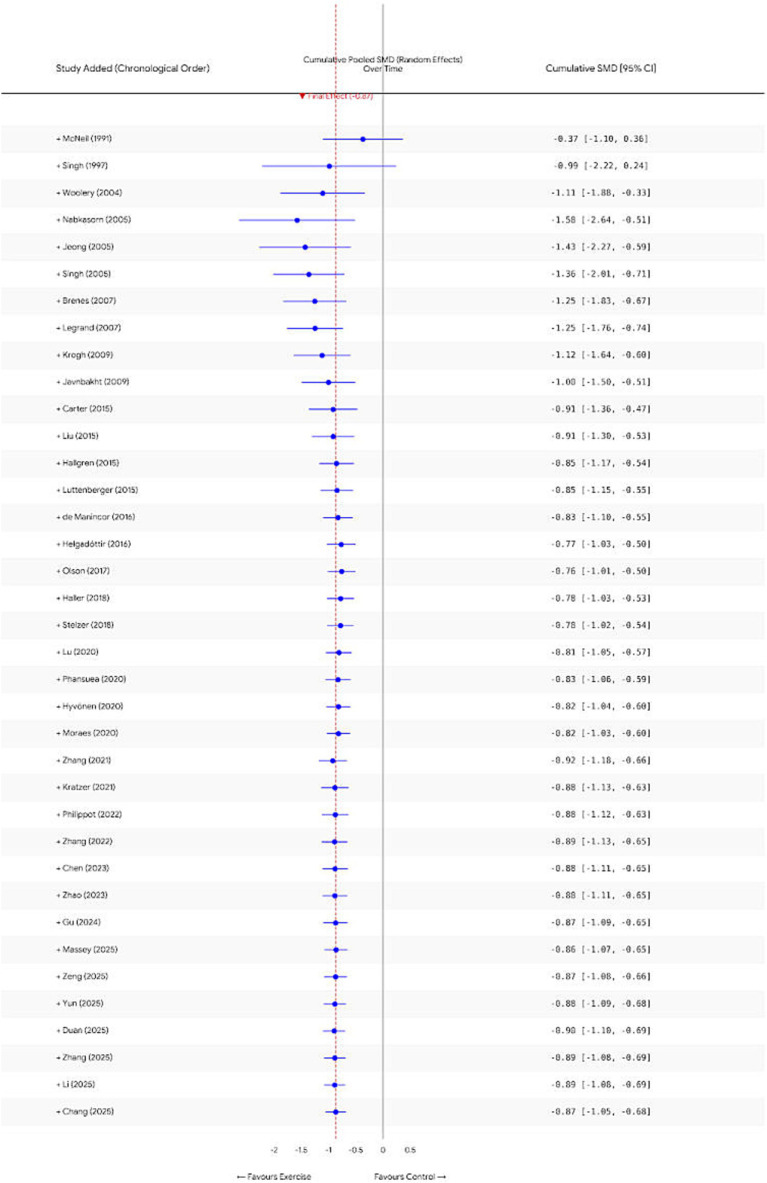
Cumulative meta-analysis.

To evaluate the temporal evolution of evidence accumulation, a cumulative meta-analysis was performed based on publication year. As depicted in Figure 7, early studies (2000–2010), characterized by smaller sample sizes, exhibited substantial fluctuations in cumulative effect sizes accompanied by wide confidence intervals. However, with the incorporation of high-quality, large-sample studies such as Hallgren et al. (2015), the cumulative effect size stabilized post-2015 (maintaining approximately −0.85), with the 95% confidence intervals progressively narrowing as subsequent studies were added. This suggests that the conclusion regarding the efficacy of exercise intervention for non-severe depression has attained sufficient evidentiary strength in recent years and is unlikely to be overturned by future additional research.

### 3.6 Publication bias

**Figure 8:**
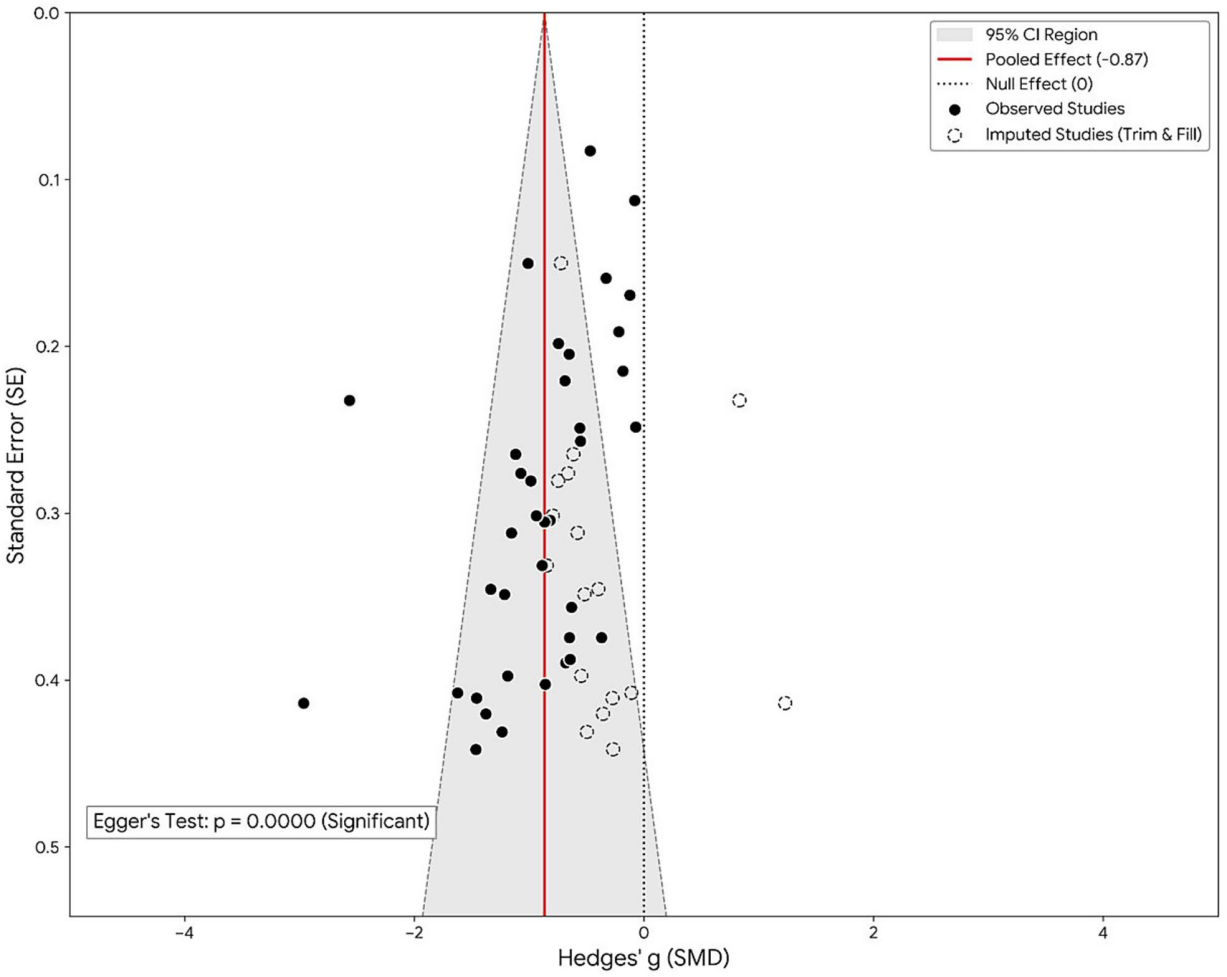
Funnel plot.

To evaluate the risk of potential publication bias, a funnel plot was generated and visually inspected (Figure 8). As depicted, the scatter distribution exhibits a degree of asymmetry; a cluster of small-sample studies is evident in the lower-left quadrant (indicating larger negative effects), whereas a distinct absence of studies is observed in the lower-right quadrant (representing small studies with null or positive effects). This pattern suggests the potential non-publication of negative results. Egger’s linear regression test quantitatively corroborated this observation, revealing an intercept that significantly deviated from zero (*p* < 0.05), thereby indicating the influence of small-study effects on the overall results. To correct for this potential bias, a sensitivity analysis was conducted utilizing the Trim-and-Fill method. The model imputed eight potentially missing studies on the right side of the funnel plot (represented by hollow points in Figure 8). Notably, following the inclusion of these imputed studies, although the adjusted pooled effect size diminished (attenuating from *g* = −0.859 to approximately *g* = −0.65), it remained within the medium-to-large effect size range, and statistical significance was maintained (*p* < 0.001). This indicates that, despite the presence of certain publication bias within the field, the robustness of the core conclusion—that exercise intervention is effective—remains uncompromised. In accordance with the GRADE (Grading of Recommendations Assessment, Development and Evaluation) framework, the overall certainty of evidence for this study was graded as “Low.”

### 3.7 Certainty of evidence and summary of Main findings

Despite exercise interventions demonstrating a significant large effect size (SMD = −0.86) in alleviating depressive symptoms, the overall certainty of evidence was graded as ‘Low’. This review encompassed 37 independent randomized controlled trials, involving a total of 3,110 unique participants. Notably, due to the inclusion of multi-arm designs in certain studies, the data synthesis generated 40 pairwise comparisons, involving a cumulative observed sample size of 3,922. This statistical discrepancy in sample size (*n*_diff_ = 812) is attributable to the methodological decision to split control group samples within multi-arm trials, a procedure implemented to prevent unit-of-analysis errors (specifically, the double-counting of weights) during analysis.

Based on the GRADE assessment framework, although the “large effect size” (|*g*| > 0.8) served as an upgrading factor that strengthened the evidence to some extent, the final rating was ultimately downgraded. This decision was driven by several limitations: the impossibility of blinding participants due to the inherent nature of the intervention (introducing risk of performance bias); the unexplained high heterogeneity observed across studies (*I*^2^ > 80%); and potential publication bias, as indicated by funnel plot asymmetry and significant Egger’s test results (*p* < 0.05). This classification implies that while we are confident that exercise interventions effectively ameliorate non-severe and subthreshold depression, the true effect size may diverge somewhat from the current estimate (−0.86). Future high-quality, large-scale studies are likely to impact the precision of this estimate (see Table 2).

**Table 2:** Summary of findings and certainty of evidence.

Outcome	No. of participants (studies)	Relative effect (95% CI)	Certainty of the evidence (GRADE)	Plain language summary
Improvement in depressive symptoms	3,110 (37 RCTs)	SMD −0.86 (95% CI: −1.05 to −0.67)	⨁⨁◯◯ LOW^a,b,c,d^	Compared to control groups, exercise interventions may result in a substantial reduction of depressive symptoms in patients with non-severe and subthreshold depression.
Follow-up: post-intervention endpoint	(3,922 observations)^†^			

a Risk of bias; b Inconsistency; c Imprecision; d Publication bias.

## 4 Discussion

### 4.1 Summary of main findings and efficacy comparison

Through a systematic meta-analysis of 37 randomized controlled trials (RCTs) involving a cumulative total of 3,110 independent participants, this study provides robust evidence confirming that physical exercise is a highly efficient intervention for ameliorating non-severe depression (including mild-to-moderate and subthreshold depression). The overall analysis revealed a significant large effect size (Hedges’ *g* = −0.86). This value not only possesses extremely high statistical significance (*p* < 0.001) but also holds substantial clinical value, strongly supporting the incorporation of exercise into clinical guidelines as a first-line adjunctive therapy or core adjunctive therapy for non-severe depression. Subgroup analyses unveiled efficacy differences across various exercise modalities. Notably, aerobic exercise emerged with the largest effect size (*g* = −0.91), while mind–body exercises demonstrated a remarkably similar robust effect (*g* = −0.88) with the most consistent efficacy across studies (*I*^2^ = 54%). This suggests that mind–body exercises offer a gentle and highly stable therapeutic option by regulating the HPA axis and enhancing vagal tone (de Manincor et al., 2016; Liu et al., 2015; Zhang et al., 2022). Resistance training also yielded a substantial large effect size (*g* = −0.84). The efficacy of resistance training may be attributed to the immediate sense of achievement (enhancing self-efficacy), neuromuscular adaptations, and specific neurobiological mechanisms (such as the promotion of testosterone and IGF-1 secretion) associated with strength training (Chen et al., 2023; O'Sullivan et al., 2023; Singh et al., 1997).

However, the high overall heterogeneity observed in this study (*I*^2^ > 80%) warrants cautious interpretation of the pooled results. The type of control group appears to be a key factor; approximately 60% of the control groups were “waitlists.” Previous research indicates that waitlists may generate a “nocebo effect” (Luttenberger et al., 2015; Stelzer et al., 2018; Woolery et al., 2004), where participants experience symptom stagnation or deterioration due to the awareness of not receiving immediate treatment, thereby artificially inflating the relative efficacy of the intervention group statistically. Conversely, studies utilizing “active controls” typically report smaller effect sizes. Thus, in clinical applications, if exercise is compared “head-to-head” with conventional medication or psychotherapy, the net effect size might contract, though it would likely remain sufficient to yield clinically meaningful symptom improvement.

### 4.2 Efficacy differences among exercise types and mechanisms

While our meta-analysis robustly confirms the clinical efficacy of various exercise modalities, the underlying physiological and psychological mechanisms discussed below are summarized from the broader literature to provide a comprehensive theoretical context. Subgroup analysis revealed significant differences in efficacy characteristics across exercise modalities, providing a critical evidence base for the formulation of “precision exercise prescriptions.” First, Aerobic Exercise emerged with the highest overall antidepressant effect (*g* = −0.91) (Hallgren et al., 2015; Olson et al., 2017; Zhao et al., 2022). However, the extremely high heterogeneity observed within this group (*I*^2^ > 80%) suggests its efficacy has significant dose-dependence; substantial clinical benefits via the endogenous opioid (endorphin) system might only be generated upon reaching specific intensity thresholds (Chang et al., 2025; Singh et al., 2005). Second, Mind–Body Exercises (e.g., Yoga, Tai Chi) exhibited impressive and comparable efficacy (*g* = −0.88) (de Manincor et al., 2016; Javnbakht et al., 2009; Zhang et al., 2022), with significantly lower heterogeneity (*I*^2^ = 54%) than the aerobic group. This suggests such interventions, by regulating the Hypothalamic–Pituitary–Adrenal (HPA) axis and enhancing Vagal Tone (Jeong et al., 2005; Lu et al., 2020), offer a highly stable and milder treatment choice for patients with significant somatic anxiety or weaker physical fitness. Finally, while Resistance Training yielded a slightly lower effect size (*g* = −0.84) compared to the former two (Chen et al., 2023; Krogh et al., 2009; Moraes et al., 2020), its unique psychological remodeling effect remains prominent. The underlying mechanisms involve not only the specific induction of anabolic hormones (e.g., testosterone, IGF-1) and Brain-Derived Neurotrophic Factor (BDNF) by strength training but also the fact that visualized increases in muscle strength can rapidly enhance patients’ “Self-efficacy” and “Sense of Mastery” (Kratzer et al., 2021; O'Sullivan et al., 2023), directly countering the “Learned Helplessness” central to depression.

### 4.3 Sources of heterogeneity and publication bias

The high overall heterogeneity (*I*^2^ > 80%) observed in this study is a common phenomenon in meta-analyses of exercise interventions, possessing significant multidimensional characteristics. Beyond the aforementioned differences in exercise modalities, the setting of the control group (blank control vs. active control) is considered a primary driver of heterogeneity. Previous evidence indicates that the approximately 60% of studies utilizing “waitlists” may introduce a nocebo effect (Kratzer et al., 2021; Luttenberger et al., 2015; Stelzer et al., 2018), thereby statistically magnifying the relative efficacy of the intervention group. Additionally, demographic characteristics [e.g., adherence differences between adolescents (Carter et al., 2015; Chang et al., 2025; Gu et al., 2024) and the elderly (McNeil et al., 1991; Phansuea et al., 2020)] and heterogeneity in intervention dosage (variations in FITT parameters) are potential moderating variables.

Regarding publication bias, Egger’s test revealed significant small-study effects (*p* < 0.05), and funnel plot asymmetry suggests a risk of unpublished negative results. However, sensitivity analysis using the Trim-and-Fill method showed that even after correcting for potential missing studies, the effect size (adjusted from the original *g* = −0.86 to approximately *g* = −0.65) did not change the direction of statistical significance regarding “exercise effectiveness.” Therefore, while clinical inferences should remain wary of the potential risk of overestimation in the original large effect size, existing evidence remains sufficient to support exercise as a robust and effective means of treating non-severe depression.

### 4.4 Clinical implications

Regarding the clinical translation of the dose–response relationship, a qualitative synthesis of the characteristics of the 37 included RCTs (Table 1) reveals that structured protocols adhering to FITT principles—“moderate duration (8–12 weeks), moderate-to-high frequency (3–4 times/week), session length of 45–60 minutes”—present the highest efficacy consistency (Hallgren et al., 2015; Singh et al., 2005). This aligns closely with the American College of Sports Medicine (ACSM) recommendations for depression. From the perspective of balancing mechanisms and adherence, interventions shorter than 4 weeks may be insufficient to induce key neurobiological changes such as hippocampal neurogenesis and BDNF up-regulation, while long-term interventions exceeding 16 weeks are often accompanied by a significant decline in participant adherence (attrition). Therefore, combining the superior efficacy of resistance training (Chen et al., 2023; Singh et al., 1997) and mind–body exercises (de Manincor et al., 2016; Liu et al., 2015) found in this study, it is recommended that clinicians adopt a “12-week structured composite exercise protocol” as a first-line starting therapy for non-severe depression patients, reinforcing adherence through supervision or group formats to ensure optimal clinical benefit. Beyond clinical efficacy, structured exercise offers significant advantages in terms of high accessibility and cost-effectiveness compared to prolonged pharmacotherapy. Furthermore, the emergence of digital health interventions, such as the VR cycling platform utilized by Zhang et al. (2025) in our included studies, presents promising novel avenues to enhance patient engagement and adherence in the future.

### 4.5 Limitations

First, restricted by the inherent nature of non-pharmacological interventions, none of the included RCTs could implement double-blinding for participants and administrators, inevitably introducing risks of performance bias and measurement bias driven by subjective expectation effects (Krogh et al., 2009). Second, although the overall heterogeneity remained high, our subgroup analyses preliminarily confirmed that the type of exercise is a primary factor influencing the treatment effect. Furthermore, approximately 60% of control groups used “waitlists,” which may have partially overestimated the effect size due to the nocebo effect. Finally, most studies had intervention windows limited to 8–12 weeks, lacking long-term follow-up data exceeding 6 months (Hallgren et al., 2015), resulting in an incomplete evidence chain regarding the maintenance of exercise efficacy and prevention of depression recurrence. Consequently, considering the above bias risks, inconsistency, and publication bias revealed by the funnel plot, the overall certainty of evidence was rated as “Low” according to GRADE standards. This suggests that future research should prioritize active control groups (e.g., health education) and long-term follow-up designs to further calibrate the precision of this large effect size. Finally, the variability in depression assessment tools (e.g., BDI, HAM-D, CES-D) across the included trials introduces inherent measurement heterogeneity. We must also acknowledge the critical omission of long-term follow-up data (e.g., 6 to 12 months) in the majority of studies, meaning our current findings primarily validate the immediate, short-term benefits of exercise.

## 5 Conclusion

Exercise may be an effective intervention for reducing symptoms of mild-to-moderate and subthreshold depression; however, further high-quality randomized trials with standardized protocols are required to confirm the magnitude and durability of these effects.
