# Supplementary material for: Can exercise truly alleviate mild-to-moderate and subthreshold depression? Evidence from randomized controlled trials
Source: Front Psychol. 2026 Apr 14;17:1815160. doi: 10.3389/fpsyg.2026.1815160 (PMC13122189; doi:10.3389/fpsyg.2026.1815160)
Supplement: Supplementary file 1 [file Data_Sheet_1.PDF]

Web of science

TS=("physical activity" OR exercise* OR sport* OR workout* OR "physical fitness" OR "aerobic exercise*" OR run* OR jog* OR walk* OR swim* OR cycl* OR "resistance train*" OR "strength train*" OR "weight lifting" OR HIIT OR "high-intensity interval" OR SIT OR "sprint interval" OR "mind-body" OR yoga OR "tai chi" OR taiji OR qigong OR pilates OR baduanjin OR "ball sport*" OR "team sport*" OR "racket sport*" OR basketball OR soccer OR football OR volleyball OR badminton OR tennis OR "table tennis" OR ping-pong OR "moderate intensity" OR "vigorous intensity" OR "low intensity") AND TS=("depressive symptom*" OR "subthreshold depression" OR "subsyndromal depression" OR "minor depression" OR "mild depression" OR "moderate depression" OR "mild-to-moderate depression" OR "non-severe depression" OR dysphoria OR "low mood") AND TS=("randomized controlled trial*" OR RCT OR "random allocation" OR "clinical trial" OR randomly) NOT TS=("major depressive disorder" OR MDD OR "bipolar disorder" OR schizophrenia OR psychosis OR PTSD OR "post-traumatic stress" OR autism OR ADHD OR "eating disorder*" OR anorexia OR bulimia OR "substance abuse" OR addiction OR cancer* OR neoplasm* OR stroke* OR "cardiovascular disease" OR "heart failure" OR HIV OR dementia OR Alzheimer* OR Parkinson* OR "multiple sclerosis" OR epilepsy OR "traumatic brain injury" OR "spinal cord injury" OR fibromyalgia OR arthritis OR osteoarthritis OR "chronic pain" OR "chronic fatigue syndrome" OR COPD OR "chronic kidney disease" OR dialysis OR "terminal illness" OR "palliative care" OR postpartum OR pregnancy OR rat OR rats OR mice OR mouse OR murine OR animal* OR dog* OR protocol* OR review* OR "meta-analysis")

pubmed

("Exercise"[Mesh] OR "Sports"[Mesh] OR "Physical Fitness"[Mesh] OR "physical activity"[tiab] OR exercise*[tiab] OR sport*[tiab] OR workout*[tiab] OR "physical fitness"[tiab] OR "aerobic exercise*"[tiab] OR run*[tiab] OR jog*[tiab] OR walk*[tiab] OR swim*[tiab] OR cycl*[tiab] OR "resistance train*"[tiab] OR "strength train*"[tiab] OR "weight lifting"[tiab] OR HIIT[tiab] OR "high-intensity interval"[tiab] OR SIT[tiab] OR "sprint interval"[tiab] OR "mind-body"[tiab] OR yoga[tiab] OR "tai chi"[tiab] OR taiji[tiab] OR qigong[tiab] OR pilates[tiab] OR baduanjin[tiab] OR "ball sport*"[tiab] OR "team sport*"[tiab] OR "racket sport*"[tiab] OR basketball[tiab] OR soccer[tiab] OR football[tiab] OR volleyball[tiab] OR badminton[tiab] OR tennis[tiab] OR "table tennis"[tiab] OR ping-pong[tiab] OR "moderate intensity"[tiab] OR "vigorous intensity"[tiab] OR "low intensity"[tiab]) AND ("depressive symptom*"[tiab] OR "subthreshold depression"[tiab] OR "subsyndromal depression"[tiab] OR "minor depression"[tiab] OR "mild depression"[tiab] OR "moderate depression"[tiab] OR "mild-to-moderate depression"[tiab] OR "non-severe depression"[tiab] OR dysphoria[tiab] OR "low mood"[tiab]) AND ("Randomized Controlled Trial"[Publication Type] OR "randomized controlled trial*"[tiab] OR RCT[tiab] OR "random allocation"[tiab] OR "clinical trial"[tiab] OR randomly[tiab]) NOT ("Animals"[Mesh:NoExp] OR

"Review"[Publication Type] OR "Meta-Analysis"[Publication Type] OR "Major Depressive Disorder"[Mesh] OR "Bipolar Disorder"[Mesh] OR "Schizophrenia"[Mesh] OR "Neoplasms"[Mesh] OR "Cardiovascular Diseases"[Mesh] OR "HIV"[Mesh] OR "major depressive disorder"[tiab] OR MDD[tiab] OR "bipolar disorder"[tiab] OR schizophrenia[tiab] OR psychosis[tiab] OR PTSD[tiab] OR "post-traumatic stress"[tiab] OR autism[tiab] OR ADHD[tiab] OR "eating disorder*"[tiab] OR anorexia[tiab] OR bulimia[tiab] OR "substance abuse"[tiab] OR addiction[tiab] OR cancer*[tiab] OR neoplasm*[tiab] OR stroke*[tiab] OR "cardiovascular disease"[tiab] OR "heart failure"[tiab] OR HIV[tiab] OR dementia[tiab] OR Alzheimer*[tiab] OR Parkinson*[tiab] OR "multiple sclerosis"[tiab] OR epilepsy[tiab] OR "traumatic brain injury"[tiab] OR "spinal cord injury"[tiab] OR fibromyalgia[tiab] OR arthritis[tiab] OR osteoarthritis[tiab] OR "chronic pain"[tiab] OR "chronic fatigue syndrome"[tiab] OR COPD[tiab] OR "chronic kidney disease"[tiab] OR dialysis[tiab] OR "terminal illness"[tiab] OR "palliative care"[tiab] OR postpartum[tiab] OR pregnancy[tiab] OR rat[tiab] OR rats[tiab] OR mice[tiab] OR mouse[tiab] OR murine[tiab] OR animal*[tiab] OR dog*[tiab] OR protocol*[tiab] OR review*[tiab] OR "meta-analysis"[tiab])

Cohrance
(physical activity OR exercise* OR sport* OR workout* OR "physical fitness" OR "aerobic exercise"* OR run* OR jog* OR walk* OR swim* OR cycl* OR "resistance training" OR "strength training" OR "weight lifting" OR HIIT OR "high intensity interval"* OR SIT OR "sprint interval"* OR "mind body" OR yoga OR "tai chi" OR taiji OR qigong OR pilates OR baduanjin OR "ball sports" OR "team sports" OR "racket sports" OR basketball OR soccer OR football OR volleyball OR badminton OR tennis OR "table tennis" OR ping pong OR "moderate intensity" OR "vigorous intensity" OR "low intensity")

Info
((exercise OR "physical activity" OR "physical fitness" OR "aerobic exercise" OR "resistance training" OR "strength training" OR "weight lifting" OR HIIT OR "high intensity interval" OR SIT OR "sprint interval" OR "mind body" OR yoga OR "tai chi" OR qigong OR pilates OR baduanjin OR "ball sports" OR "team sports" OR "racket sports" OR basketball OR soccer OR football OR volleyball OR badminton OR tennis OR "table tennis" OR "ping pong" OR "martial arts" OR "judo" OR "karate" OR "climbing" OR "exergaming" OR "moderate intensity" OR "vigorous intensity") AND ("depressive symptoms" OR "subthreshold depression" OR "subsyndromal depression" OR "minor depression" OR "mild depression" OR "moderate depression" OR "mild to moderate depression" OR "non-severe depression" OR dysphoria OR "low mood") AND ("randomized controlled trial" OR RCT OR "random allocation" OR "clinical trial" OR randomly)) NOT (MDD OR "major depressive disorder" OR "bipolar disorder" OR schizophrenia OR psychosis OR PTSD OR "post-traumatic stress" OR autism OR ADHD OR "eating disorder"

OR anorexia OR bulimia OR "substance abuse" OR addiction OR cancer* OR neoplasm* OR stroke* OR "cardiovascular disease" OR "heart failure" OR HIV OR dementia OR Alzheimer* OR Parkinson* OR "multiple sclerosis" OR epilepsy OR "traumatic brain injury" OR "spinal cord injury" OR fibromyalgia OR arthritis OR osteoarthritis OR "chronic pain" OR "chronic fatigue syndrome" OR COPD OR "chronic kidney disease" OR dialysis OR "terminal illness" OR "palliative care" OR postpartum OR pregnancy OR rat OR rats OR mice OR mouse OR animal* OR dog* OR protocol* OR review* OR "meta analysis")

Embase
('physical activity' OR exercise* OR sport* OR workout* OR 'physical fitness' OR 'aerobic exercise*' OR run* OR jog* OR walk* OR swim* OR cycl* OR 'resistance training' OR 'strength training' OR 'weight lifting' OR HIIT OR 'high intensity interval*' OR SIT OR 'sprint interval*' OR 'mind body' OR yoga OR 'tai chi' OR taiji OR qigong OR pilates OR baduanjin OR 'ball sports' OR 'team sports' OR 'racket sports' OR basketball OR soccer OR football OR volleyball OR badminton OR tennis OR 'table tennis' OR 'ping pong' OR 'martial arts' OR judo OR karate OR climbing OR exergaming OR 'moderate intensity' OR 'vigorous intensity'):ti,ab,kw AND ('depressive symptom*' OR 'subthreshold depression' OR 'subsyndromal depression' OR 'minor depression' OR 'mild depression' OR 'moderate depression' OR 'mild to moderate depression' OR 'non-severe depression' OR dysphoria OR 'low mood'):ti,ab,kw AND ('randomized controlled trial*' OR RCT OR 'random allocation' OR 'clinical trial' OR randomly):ti,ab,kw NOT ('major depressive disorder' OR MDD OR 'bipolar disorder' OR schizophrenia OR psychosis OR PTSD OR autism OR ADHD OR 'eating disorder*' OR cancer* OR stroke* OR HIV OR dementia OR Alzheimer* OR Parkinson* OR rat OR rats OR mice OR animal* OR protocol* OR review* OR 'meta analysis'):ti,ab,kw
