# Supplementary figures and images for: Can exercise truly alleviate mild-to-moderate and subthreshold depression? Evidence from randomized controlled trials
Source: Front Psychol. 2026 Apr 14;17:1815160. doi: 10.3389/fpsyg.2026.1815160 (PMC13122189; doi:10.3389/fpsyg.2026.1815160)

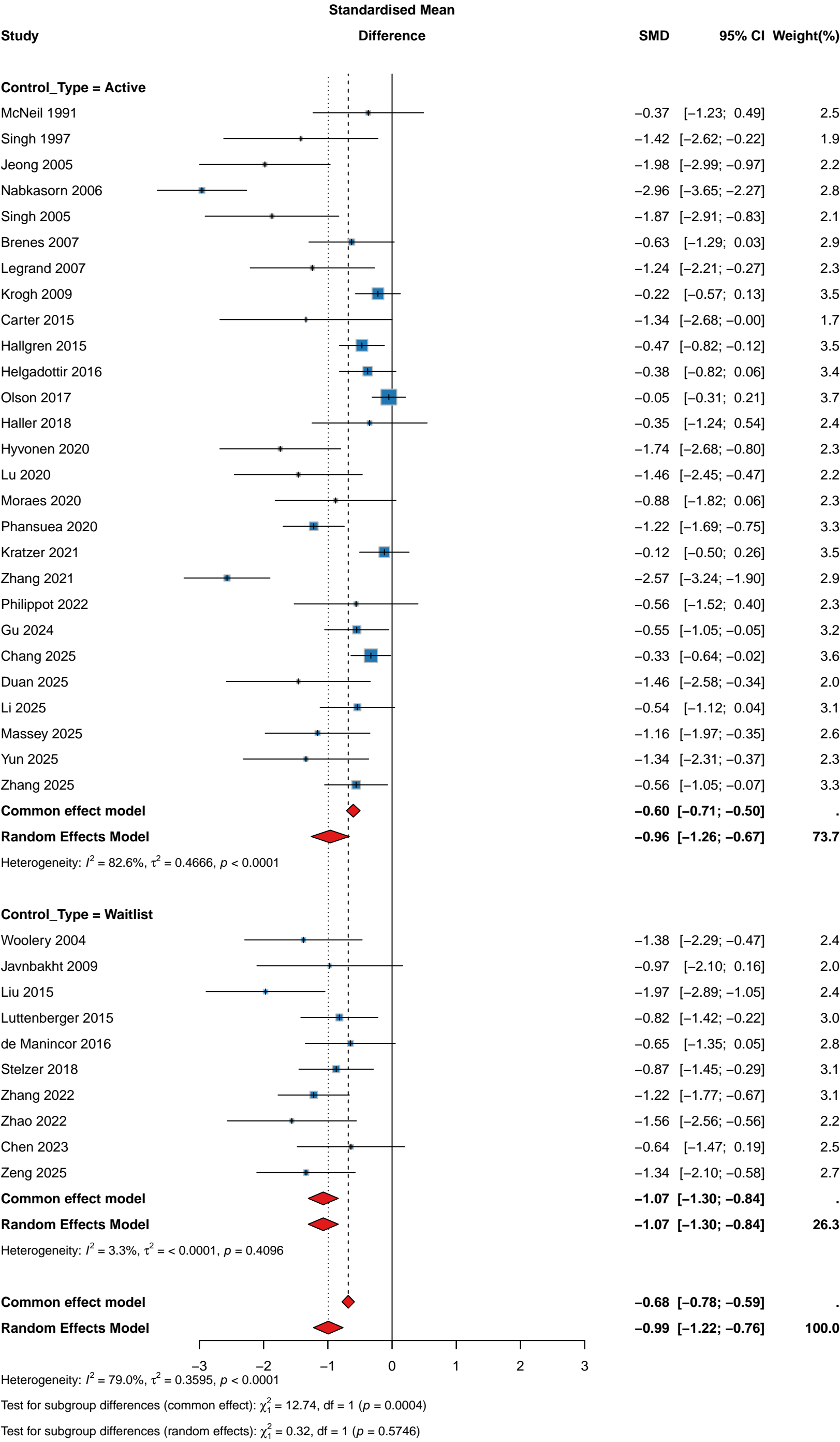

Supplement: Supplementary file 2 [file Data_Sheet_2.PDF]
